# Prognostic value of iron-metabolism biomarkers in critically ill patients with atrial fibrillation: a machine learning-based retrospective cohort study

**DOI:** 10.1097/JS9.0000000000002750

**Published:** 2025-06-12

**Authors:** Chaoqun Huang, Shangzhi Shu, Xuejun Hui, Megumi Narisawa, Xiongjie Jin, Shuyan Li, Xian Wu Cheng

**Affiliations:** a Department of Cardiology, the First Hospital of Jilin University, Changchun, China; b Department of Nephrology, the Second Hospital of Jilin University, Changchun, China; c Department of Cardiology, Nagoya University Graduate School of Medicine, Nagoya, Japan; d Department of Cardiology and Hypertension, Jilin Provincial Key Laboratory of Stress and Cardiovascular Disease, Yanbian University Hospital, Yanji, China

**Keywords:** atrial fibrillation, critically ill patients, iron metabolism, machine learning, mortality prediction, prognosis

## Abstract

Iron homeostasis may significantly impact outcomes in critically ill patients with atrial fibrillation (AF), yet its prognostic value remains unclear. Analyzing 2145 AF patients from MIMIC-IV database, we found 43% mortality (n = 928) at 1-year follow-up. Elevated ferritin (Q4 HR 1.59, 95%CI 1.30–1.95, *P* < 0.001) and serum iron (Q4 HR 1.33, 95%CI 1.11–1.60, *P* = 0.002) predicted higher mortality, while higher transferrin (Q4: HR 0.65, 95%CI: 0.53–0.79, *P* < 0.001) and TIBC (Q4: HR 0.64, 95%CI: 0.53–0.79, *P* < 0.001) were protective. The gradient boosting decision trees model achieved excellent prediction (AUC 0.85 training/validation, 0.71 testing), with TIBC as the top predictor. These iron-metabolism indicators provide strong prognostic value for risk stratification in critically ill patients with AF.

## Introduction

Atrial fibrillation (AF) is the most common sustained cardiac arrhythmia. The occurrence of AF in critically ill patients is associated with significantly elevated risks of morbidity and mortality^[^1^]^, underscoring the need for effective AF management and accurate risk stratification.

Iron metabolism plays a pivotal role in cardiovascular health and disease^[^2^]^. High ferritin and iron intake were associated with an increased risk of AF^[^3,4^]^. However, the prognostic value of iron makers in critically ill patients with AF remains poorly understood. The goals of this study were to clarify iron metabolism’s prognostic role in critically ill patients with AF and develop a machine learning-enhanced risk stratification tool to identify high-risk subgroups. This cohort study has been reported in line with the STROCSS guidelines^[^5^]^.

**HIGHLIGHTS**

- Iron homeostasis (particularly TIBC) independently predicts mortality in critically ill patients with AF.
- Machine learning integration of iron markers achieves superior prediction (AUC 0.85) versus conventional scores.
- Dual-directional effects: Ferritin (↑risk) and TIBC/transferrin (↓risk) provide complementary prognostic value.

## Methodology

**Figure 1.**
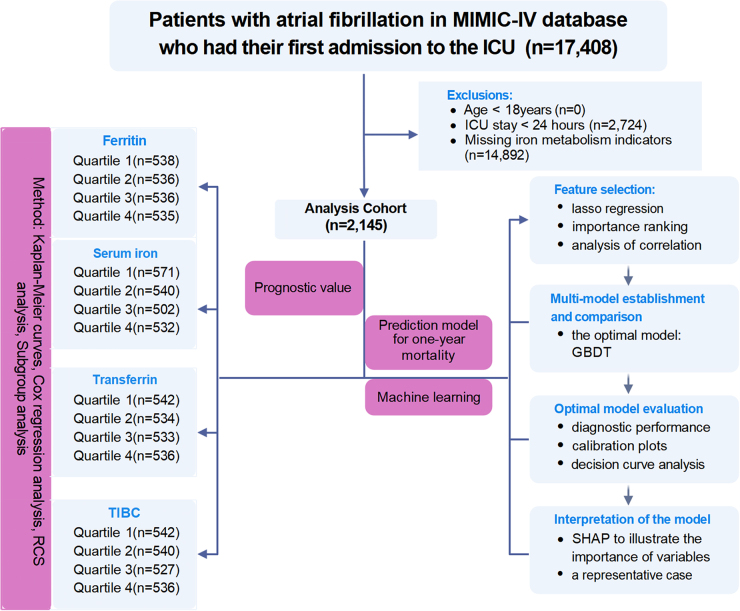
Patient screening flow from the MIMIC-IV database.

This single-center retrospective cohort study drew its data from the MIMIC-IV. AF was identified using ICD-9 code (427.31) and ICD-10 codes (I48.91, I48.0, I48.2, I48.19, I48.20, I48.1, I48.21, and I48.11). The prognostic values of the iron-metabolism indicators (ferritin, serum iron, transferrin, and total iron-binding capacity [TIBC]) were analyzed by quartiles using Kaplan-Meier, multivariable Cox regression, and a restricted cubic spline (RCS) analysis. For mortality prediction, feature selection combined LASSO regression and Boruta algorithm to identify key predictors. Machine learning (ML) models were developed, with performance assessed by AUC, calibration curves, and decision curve analysis. The optimal model was interpreted via SHAP values to evaluate feature importance. Continuous variables are presented as mean ± SD or median[IQR], categorical variables as n(%), analyzed using appropriate statistical tests (t-test/Mann-Whitney U-test or χ^2^-test) with significance at *P* <0.05. The study protocol was registered in the Research Registry (UIN: researchregistry11251; available at: https://www.researchregistry.com/browse-the-registry#home/). The study flowchart is shown in Fig. 1. Methodological details are provided in the Supplemental Digital Content.

## Results

This study included 2145 critically ill patients with AF. The patients were divided into survival and death groups based on their 1-year outcomes after ICU admission, with 928 (43%) patients succumbing. Baseline characteristics are summarized in Table S1 (available at: http://links.lww.com/JS9/E397). Notably, the death group exhibited significantly higher ferritin levels (*P* < 0.001) and lower transferrin/TIBC levels (both *P* < 0.001), while serum iron showed no intergroup difference.

### The prognostic values of the iron-metabolism indicators

**Figure 2.**
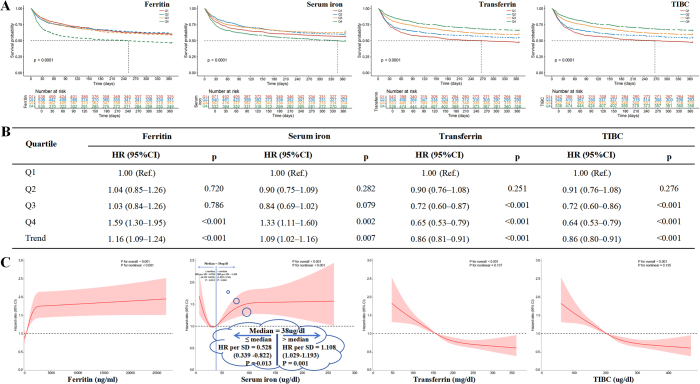
The prognostic values of the iron-metabolism indicators. (A) The relationship between the iron-metabolism indicators at different levels and all-cause mortality, analyzed using Kaplan-Meier curves. (B) The Cox proportional hazard ratios for the iron-metabolism indicators. Adjusted for RBC, HGB, RDW, HCT, NLR, SOFA score, gender, age, hypertension, diabetes, heart failure, myocardial infarction, sepsis, AKI. (C) The restricted cubic spline (RCS) analysis of iron-metabolism indicators with all-cause mortality. Adjusted for RBC, HGB, RDW and HCT. AKI: acute kidney injury, HCT: hematocrit, HGB: hemoglobin, NLR: neutrophil-to-lymphocyte ratio; RBC: red blood cells, RDW: red blood cell distribution width, SOFA: sequential organ failure assessment; TIBC: total iron-binding capacity.

Quartile stratifications of iron-metabolism indicators were established (Table S2, available at: http://links.lww.com/JS9/E397), with these thresholds used for subsequent analyses. Kaplan-Meier analysis demonstrated significantly worse survival for patients with elevated ferritin and reduced transferrin/TIBC levels (all log-rank *P* < 0.001), while serum iron exhibited a U-shaped mortality relationship (Fig. 2A). Then, multivariable Cox models (fully adjusted) confirmed iron-mortality associations: ferritin (Q4: Q4: HR 1.59, 95%CI: 1.30–1.95, *P* < 0.001) and serum iron (Q4: HR 1.33, 95%CI: 1.11–1.60, *P* = 0.002) predicted increased risk, while transferrin (Q3: HR 0.72, 95%CI: 0.60–0.87, *P* < 0.001; Q4: HR 0.65, 95%CI: 0.53–0.79, *P* < 0.001) and TIBC (Q3: HR 0.72, 95%CI: 0.60–0.86, *P* < 0.001; Q4: HR 0.64, 95%CI: 0.53–0.79, *P* < 0.001) showed protection (Fig. 2B and Table S3, available at: http://links.lww.com/JS9/E397).

RCS analyses revealed distinct iron-mortality associations: ferritin was negatively associated, serum iron exhibited a U-shaped relationship, and transferrin and TIBC showed positive associations (Fig. 2C). Serum iron levels below the median (38 µg/dL) were associated with an improved prognosis (HR per SD 0.53, 95%CI: 0.34–0.82, *P* = 0.013), whereas levels above the median were linked to adverse outcomes (HR per SD 1.11, 95%CI: 1.03–1.19, *P* = 0.001).

To validate robustness, we dichotomized iron-metabolism indicators at median values and performed stratified analyses. The results showed higher ferritin and lower transferrin/TIBC levels were consistently associated with increased long-term mortality across all subgroups (Figure S1, available at: http://links.lww.com/JS9/E396).

In addition, higher ferritin and lower transferrin/TIBC levels were associated with longer hospital and ICU stays (all *P* < 0.001), whereas serum iron levels showed no significant association (Figure S2, available at: http://links.lww.com/JS9/E396).

### The prediction model of 1-year all-cause mortality

Feature selection via LASSO and Boruta algorithms initially identified 18 potential predictors, which were refined to 16 after excluding transferrin and neutrophils due to high collinearity (TIBC-transferrin: r = 1.0; WBC-neutrophils: r = 0.79; both *P* < 0.001) (Table S4, available at: http://links.lww.com/JS9/E397and Figure S3, available at: http://links.lww.com/JS9/E396).

**Figure 3.**
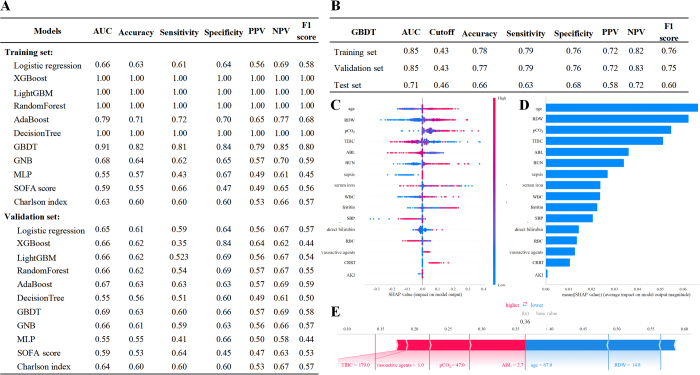
Multi-model comparison and optimal model evaluation. (A) The predictive performance of multiple machine learning models for 1-year mortality prediction. (B) The diagnostic performance of the optimal GBDT model. (C) Feature attributions in SHAP analysis of the optimal GBDT model, where each line represents a feature, with SHAP values plotted on the x-axis. Red dots: higher values, blue dots: lower values. (D) Variable importance in SHAP analysis of the optimal GBDT model shown as bars, illustrating their contribution to the model predictions. (E) SHAP scores highlighting the predicted risk of 1-year all-cause mortality for an individual subject. ABL: Serum albumin, AKI: acute kidney injury, BUN: blood urea nitrogen, CRRT: continuous renal replacement therapy, GBDT: gradient boosting decision trees, GNB: Gaussian naïve Bayes, MLP: multilayer perceptron, NPV: negative predictive value, PPV: positive predictive value, pCO2: partial pressure of carbon dioxide, RDW: red cell distribution width, RBC: red blood cells, SBP: systolic blood pressure, SOFA: sequential organ failure assessment, TIBC: total iron-binding capacity, WBC: white blood cells.

Through comprehensive evaluation of nine ML models using 10-fold cross-validation (Fig. 3A and Figure S4, available at: http://links.lww.com/JS9/E396), gradient boosting decision trees (GBDT) demonstrated optimal validation performance (AUC 0.85) with superior stability compared to eXtreme gradient boosting (suggesting potential overfitting), and was therefore selected as the final model. Notably, GBDT significantly outperformed conventional prognostic scores (SOFA score and Charlson index).

The final GBDT model, developed with 5-fold nested cross-validation (30% test set allocation), demonstrated strong discrimination: training AUC 0.85 (95%CI 0.83–0.87), validation AUC 0.83 (95%CI 0.80–0.89), and test AUC 0.71 (95%CI 0.67–0.75) (Fig. 3B and Figure S5, available at: http://links.lww.com/JS9/E396). The SHAP analysis elucidated the contribution of each variable in the GBDT model. Each feature’s contribution is shown by colored dots, with red denoting high-risk values and blue representing low-risk values (Fig. 3C). Figure 3D ranks their relative importance in the predictive model, with age, RDW, and pCO_2_ emerged as the factors with the greatest influence on prognosis. TIBC followed closely behind, while serum iron and ferritin were of moderate significance, highlighting the pivotal role of iron-metabolism indicators in predicting long-term mortality. The representative case of critically ill patients with AF is provided in Fig. 3E to demonstrate the interpretability of the model: the patient achieved a low SHAP prediction score (0.36) and survived at 1 year following ICU admission.

## Discussion

Iron is a vital mineral in the human body, playing a key role in numerous physiological processes, such as oxygen transport and storage, enzyme activity, and mitochondrial function^[^2^]^. Several studies have shown that even in the absence of anemia, iron directly affects non-hematopoietic tissues that are essential for physical performance, such as skeletal muscle and the myocardium^[^6,7^]^. However, when transferrin becomes highly saturated, excess iron binds to low-molecular-weight compounds, forming non-transferrin-bound iron (which is readily absorbed by hepatocytes and cardiomyocytes), potentially causing oxidant-mediated damage and leading to tissue injury and disease^[^8,9^]^. Consequently, iron dysregulation, including both iron deficiency and iron overload, is associated with the development of numerous diseases.

Iron homeostasis also plays a critical role in cardiac arrhythmias. Studies of both mice and humans have demonstrated that chronic iron overload is implicated in cardiac conduction block and AF^[^2^]^. The underlying mechanism may involve iron displacing calcium from its intracellular binding sites, thereby preventing the inactivation of calcium-dependent channels and prolonging the calcium influx^[^10^]^. This disruption of cardiac conduction can lead to the development of arrhythmias.

The present study revealed that elevated ferritin and serum iron significantly increased mortality risk in critically ill patients with AF, likely reflecting iron overload-induced oxidative stress and inflammation. In contrast, elevated transferrin and TIBC levels conferred protection, possibly by binding free iron, indicating efficient iron utilization and adequate iron reserves.

In summary, our findings further demonstrate the prognostic impact of disrupted iron metabolism on critically ill patients with AF, and they strongly support the use of iron-metabolism indicators as clinical prognostic markers. However, several limitations should be considered: (1) the single-center design limited our sample size; (2) potential unmeasured confounders (e.g., undocumented comorbidities or treatments) may persist; and (3) the causal relationship between iron dysregulation and outcomes remains unclear – whether iron supplementation or absorption inhibition improves prognosis requires further investigation.

## Conclusion

Iron-metabolism indicators, particularly TIBC, were demonstrated to be significant prognostic factors in critically ill patients with AF, providing valuable insights into their complex interplay with AF. The machine learning-based prediction model developed herein and incorporating these indicators provides a promising tool for improving the prognostic assessment and for guiding the clinical management in this vulnerable population.

## Data Availability

The datasets provided in this study can be obtained from the MIMIC-IV official website (https://physionet.org/content/mimiciv/1.0/).

## Research registration unique identifying number (UIN)

UIN: researchregistry11251; available at: https://www.researchregistry.com/browse-the-registry#home/.
